# Recurrent pancreatitis and sepsis in glycogen storage disease type Ia caused by complex heterozygous mutations in 2 sisters: Case report

**DOI:** 10.1097/MD.0000000000032510

**Published:** 2022-12-30

**Authors:** Qin Liu, Fang Yu, Huilin Lu, Jian Luo, Ting Sun, Lu Yu, Shenglian Gan

**Affiliations:** a Department of Endocrinology and Metabolism, The First People’s Hospital of Changde City, Changde, Hunan, China.

**Keywords:** glycogen storage disease type Ia, hypertriglyceridemia, pancreatitis, portal hypertension, sepsis

## Abstract

Glycogen storage disease (GSD) is a glycogen metabolism disorder caused by congenital enzyme defects, with type I being the most common. Owing to the rarity of glycogen storage disease type Ia (GSD Ia) and the involvement of diverse systems, patients are prone to delayed diagnosis and inappropriate treatment. Additional studies are required to standardize the diagnosis and treatment of GSD Ia.

**Patient concerns:**

We report 2 cases of GSD Ia that occurred in 2 sisters. The elder sister also had recurrent pancreatitis, and the pancreatic pseudocyst rupture resulted in sepsis, portal hypertension, and splenic infarction. The younger sister had the same mutation site, but the clinical phenotypes were not identical.

**Diagnosis:**

Abdominal computed tomography and laboratory examinations revealed regional portal hypertension, splenic infarction, and sepsis in the elder sister; diagnosis was confirmed by whole exome sequencing. Sanger sequencing was used to confirm that the younger sister and their parents also had the mutation site.

**Interventions:**

The elder sister was treated with corn starch therapy, and medication for antiinfection and reducing hypertriglyceridemia, inhibiting trypsin activity, relieving hyperuricemia. The younger sister was treated with raw cornstarch-based nutritional therapy and sodium bicarbonate.

**Outcomes:**

The elder sister’s infection was controlled and she gradually returned to a normal diet. After discharge, hyperlipidemia was not controlled satisfactorily, but hypoglycemia, hyperuricemia, hyperlactatemia, and anemia improved.

**Lessons:**

GSD should be considered in childhood patients with hypoglycemia, hypertriglyceridemia, hyperuricemia, and hyperlactatemia. Gene sequencing can enable quick identification of GSD subtypes. This case report highlights the common clinical manifestations can be linked to rare diseases. Clinical work requires careful observation of the correlations between patient history, physical examinations, and laboratory examinations.

## 1. Introduction

Glycogen storage disease (GSD) is a group of glycogen metabolism disorders caused by congenital enzyme defects. There are 12 known types, of which type I is the most common, with an overall incidence of approximately ~ 1/100,000. G6P hydrolase deficiency is involved in the pathogenesis of glycogen storage disease type Ia (GSD Ia). The G6PC gene encoding a hydrolase is located on chromosome 17q21. Patients with GSD Ia have a wide spectrum of clinical manifestations, including hepatomegaly, hypoglycemia, lactic acidemia, hyperlipidemia, hyperuricemia, and growth retardation.^[1,2]^ Due to the rarity of the disease, clinicians generally do not associate these common metabolic features with GSD Ia, meaning that patients are prone to missed diagnoses.

Hypertriglyceridemia (HTG) due to GSD I can induce pancreatitis, and several cases have been reported.^[3,4]^ However, severe cases of recurrent pancreatitis combined with portal hypertension, splenic infarction, and sepsis have not been reported in the past, indicating the serious consequences of GSD Ia without proper treatment.

## 2. Case report

A 20-year-old female patient presented with acute persistent pain in the upper abdomen accompanied by nausea, vomiting, and fever. Abdominal computed tomography (CT) performed at another hospital showed rupture of a pancreatic pseudocyst and massive ascites, and she was hospitalized in the intensive care unit at our hospital in April 2022.

The patient had been birthed via a full-term vaginal delivery with presentation of the head and loud crying at birth without cyanosis and asphyxia. Her mother had no special medication history during pregnancy. At 7 months of age, abdominal distention and hepatomegaly were detected. The patient began walking at 1 year of age, was breastfed until more than 1 year of age, and was shorter than other girls of her age. Frequent nosebleeds began to appear at the age of 5 years, and her skin was prone to ecchymosis. She was repeatedly treated in other hospitals for hepatomegaly, but the etiology was not clear. She had a history of 4 episodes of pancreatitis and was hospitalized several times, once in 2017, when she was housed in the pediatric intensive care unit of our hospital with hyperlipidemia (triglyceride 51.12 mmol/L), anemia (hemoglobin 92 g/L), and hyperuricemia (uric acid 616 µmol/L), and was treated with hemoperfusion, meropenem for antiinfection, and bezafibrate for lowering lipids, blood transfusion, and correction of acidosis. The patient’s pancreatitis improved after treatment and she was discharged from the hospital without reexamination and follow-up. The patient had a short stature and a baby’s face (current height 146 cm, weight 40 kg). Her vital signs were normal, her liver was enlarged and a palpable 5 cm below the costal margin, and she was prone to hunger and sweating, with poor physical strength and poor academic performance.

During this hospitalization, laboratory tests revealed blood biochemical results as follows: amylase 126 U/L (reference values 35–135), triglyceride 8.23 mmol/L (reference values 0.23–1.7), total cholesterol 5.04 mmol/L (reference values 2.23–5.17), glucose 2.4–18.1 mmol/L (reference values 3.5–6.1), and uric acid 829 umol/L (reference values 180–440). Arterial blood gas analysis revealed lactic acidosis (PH 7.31, BE −14.3 mmol/L and lactic acid 6.8 mmol/L) and hemoglobin levels of 70 g/L (reference values 115–150). Infection markers were significantly elevated: procalcitonin 61.14 ng/mL (reference values 0–0.5), high-sensitivity C-reactive protein 285.05 mg/L (reference values 0–25), IGF-1 142.2 ng/mL (reference values 83–456), −1.4 SD. Urinary protein, electrolytes, and liver function were normal. Abdominal paracentesis collected a yellowish brown purulent fluid.

**Figure 1.**
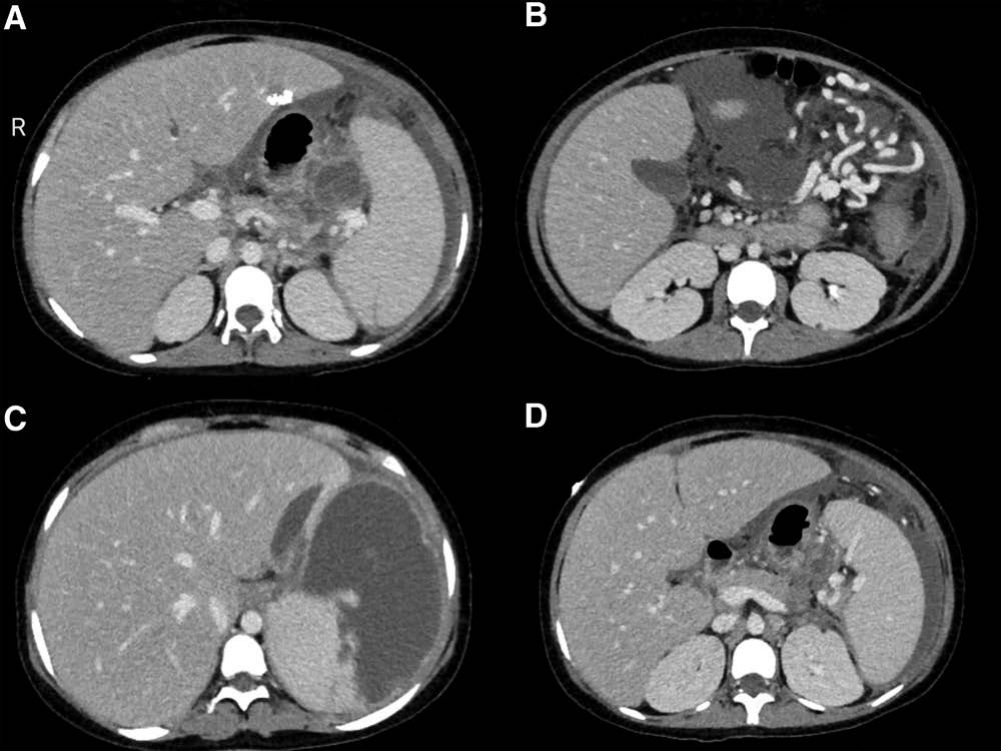
Abdominal CT of the proband: (a) Enlargement of liver and spleen. (b) Multiple varices in the left upper abdomen. (c) Cystic mass in the anterior part of the spleen.(d) The changes of chronic pancreatitis, suspected splenic vein obstruction. CT = computed tomography.

Abdominal CT exhibited (Figure 1):

1. A cystic mass in the anterior part of the spleen, multiple varices in the left upper abdomen, and massive abdominal and pelvic fluid collection, considering the possibility of regional portal hypertension (RPH) and splenic infarction.
2. Enlargement of liver and spleen;
3. Morphological changes of pancreas, possibly caused by chronic pancreatitis.

Based on the patient’s multiple metabolic disorders, whole-exome sequencing was performed, revealing 2 distinct mutations: c.648G > T (p.Leu216=) inherited from the mother and c.356A > T (p.His119Leu) was inherited from the father. Disease associated c.648G > T mutation frequencies of G6PC are present in 85–88% of Japanese patients and 36 to 40% of Chinese patients.^[1]^This complex heterozygous mutation has been reported in Chinese Taiwanese patients.^[5,6]^ The patient was treated as follows: For sepsis, abdominal puncture and drainage were performed, and the patient was given meropenem 1.0 q8h, magnesium sulfate solution administered orally 33 ml qd, somatostatin to reduce the secretion of pancreatic enzymes. Nutritional support was also provided to maintain water and electrolyte balance. To reduce hyperlipidemia in patients with metabolic disorders, fenofibrate tablets 0.2 g qd, and sodium bicarbonate tablets 1.0g tid were administered. Because HLA-B*5801 gene testing was positive, febuxostat tablets 40 mg qd were administered to relieve hyperuricemia. The patient’s condition improved and she was discharged after treatment.

**Figure 2.**
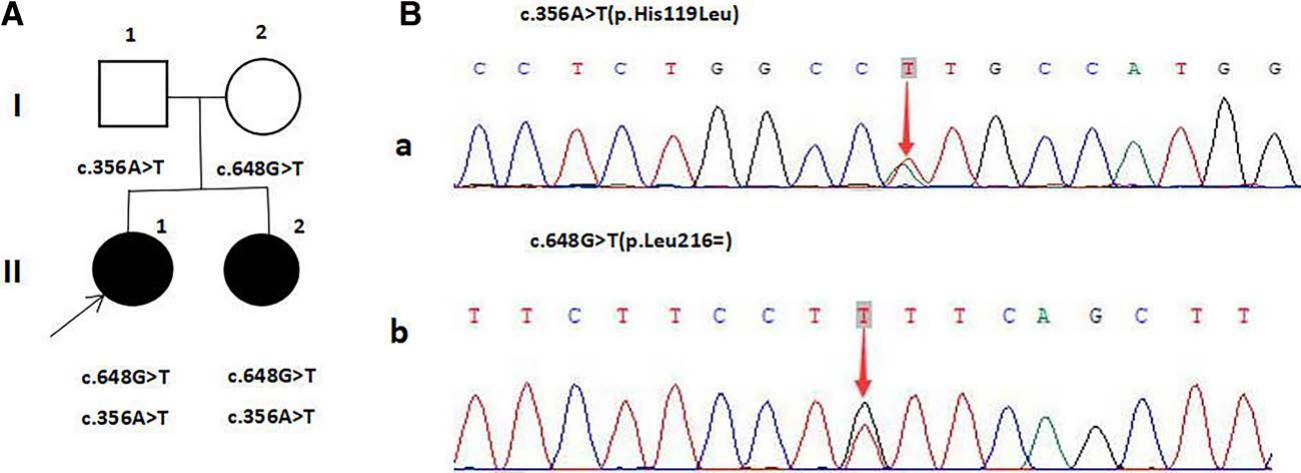
(A) Pedigree diagram of the patient with GSD Ia and her family. Affected individuals are denoted by solid symbols and unaffected individuals are denoted by open symbols. The index patient is indicated by an arrow. (B) Sanger sequencing for the younger sister: (a) c.356A>T forward sequencing (b) c.648G>T forward sequencing.

We learned that the patient’s sister had growth retardation, so we recommended that she undergo evaluations. Directly after hospitalization in May 2022, the patient’s sister was also found to be of short stature with a baby face (height 104 cm, weight 18 kg). Laboratory tests revealed blood biochemical results as follows: triglyceride 17.12 mmol/L (reference values 0.23–1.7), total cholesterol 8.95 mmol/L (reference values 2.23–5.17), fasting blood glucose 3.04 mmol/L (reference values 3.5–6.1), and uric acid 380 umol/L (reference values 180–440). Arterial blood gas analysis revealed lactic acidosis (PH 7.39, HCO 3–19.1 mmol/L, BE −4.7, and lactic acid 8.2 mmol/L). hemoglobin 98 g/L (reference values 118–156), IGF-1 27.6 ng/mL (reference values 99–483), −2.7 SD. The younger sister’s bone age was only about 2 years. Urinary protein, electrolyte levels, blood ketone, and blood ammonia were normal. Ultrasonography of the abdomen revealed hepatomegaly. Sanger sequencing confirmed that she had the same gene mutation as her sister (Fig. 2).Their father and mother were heterozygous for c.356A > T (p.His119Leu) and c.648G > T (p.Leu216=), respectively, and their clinical phenotypes were normal. Because the younger sister was only 9 years old, she was discharged from the hospital with only raw cornstarch-based nutritional therapy and treatment to correct acidosis due to limited indications for medication.

We will continue to follow-up with the 2 sisters and provide genetic counseling to the family.

### 2.1. 3. Discussion

The patient was diagnosed with GSD Ia and developed recurrent pancreatitis with portal hypertension and splenomegaly with severe infection. Genetic testing showed that a complex heterozygous mutation in the pathogenic gene G6PC c.648G>T (p.Leu216=)/ c.356A>T (p.His119Leu).

Hypoglycemia after a short fast is a major metabolic phenotype of GSD Ia. The main gluconeogenic organ involved in regulation of blood glucose homeostasis between meals is the liver. As blood glucose fall between meals, an increase in cytoplasmic glucose‐6‐phosphatase (G6P) produced in the terminal step of gluconeogenesis and glycogenolysis in the liver, kidney, and intestine is transported by G6PT into the endoplasmic reticulum, where it is hydrolyzed by G6Pase-α to make glucose, which is released into the blood. The defective G6Pase-α/G6PT complex impairs this process, creating elevated levels of cytoplasmic G6P and failing to maintain blood glucose homeostasis. Elevated cytoplasmic G6P levels drive glycogen accumulation, leading to hepatomegaly and nephromegaly, which may be further exacerbated by accumulation of neutral lipids in the liver. Other major metabolic consequences of elevated G6P levels are hyperlipidemia, hyperuricemia, and lactic acidemia^[7,8]^ the incidence of hepatic tumor is increased in patients with poor metabolic control and persistent HTG.^[9]^

Acute pancreatitis (AP) is one of the most common digestive system emergencies worldwide. In recent years, some studies have shown that hyperlipidemia surpassed alcohol is the second major cause of AP,^[10,11]^ and patients diagnosed with GSD Ia usually have obvious HTG and other clinical features. A fundamental reason for the missed diagnosis of this patient in the past was a lack of understanding of the disease.

Pancreatic RPH is the most common cause of RPH, and is mainly caused by splenic vein thrombosis or increased splenic vein pressure caused by pancreatic diseases. The incidence of splanchnic vein thrombosis (SVT) associated with AP is approximately 1to 24%, of these, about 11.2% are related to the splenic vein, and if severe can result in liver failure, portal hypertension, and spleen and intestinal necrosis_._^[12]^ The use of anticoagulants in SVT associated with AP is controversial. The rate of recanalization after anticoagulant therapy is 14% and the recanalization rate without anticoagulant therapy is 11%.^[13]^ There is insufficient evidence here to prove the effectiveness of anticoagulation in treatment of pancreatitis combined with SVT. Some researchers have found that after portal vein and SVT, anticoagulant therapy does not improve the recanalization rate, but increases the incidence of bleeding.^[14]^ Considering that this patient did not have manifestations of intestinal ischemia, we did not use anticoagulation therapy.

The diagnosis of GSD Ia is primarily based on genetic testing. Liver biopsies are rarely performed. Multiple variants have been reported in the human Gene Mutation database (http://www.hgmd.cf.ac.uk), with significant ethnic differences. c.648G>T and c.248G>A were the most common variants in Chinese patients. Patients with only one pathogenic mutation of G6PC may have one or more exons, introns, or large fragments of G6PC deletion/duplication.^[1]^ The patients in our case carried c.648G > T (p.Leu216=) as a synonymous mutation in the coding region of G6PC. According to the gnomAD database, a large-scale population frequency database, 22 heterozygous individuals with this mutation were reported, and no homozygous individuals were reported. Cell transfection assay and PT-PCR assay showed that the normal splicing product was not detected in the mutant cells.^[15]^ c.356A>T (p.His119Leu) was a missense variation in the coding region of the G6PC gene. In gnomAD, one heterozygous individual was reported, and no homozygous individuals were reported. Transfection experiments showed that the mutation results in a decrease in G6P enzyme activity.^[16]^

Management of GSD Ia requires comprehensive management, especially specialist guidance and caregiver awareness of the disease, ensuring the slow release of glucose in the intestine, which helps to keep blood glucose stable for a longer period of time. Dietary nutritional therapy based on raw cornstarch is the basic treatment for GSD Ia. The proband patient began to take 80 g raw cornstarch 4 times a day (9:00 am, 15:00 pm, 21:00 pm, 03:00 am) after her appetite improved following discharge. The patient took capillary blood glucose tests which revealed occasional episodes of hypoglycemia. However, use of 24-h continuous glucose monitoring could be a better option. Regarding the treatment of hyperlipidemia. In addition to being associated with pancreatitis, HTG may also be associated with the development of hepatocellular adenomas.^[17]^ The proband’s lipid levels were poorly controlled despite treatment with fenofibrate, and her mother reported that she did not control her fat intake. After communicating with her, the patient began adhering to a low-fat diet and regular medication. Her younger sister was not taking lipid-lowering medications because of her age, and her lipid profile was worrisome. Treatment of HTG in children still faces great challenges. For example, fenofibrate is not permitted for children. At subsequent follow-up, with the consent of the parents, we decided to also use fenofibrate for treatment of the younger sister’s hyperlipidemia. The patient’s hyperuricemia, hyperlactatemia, and anemia improved. The cause of short stature in patients with GSD Ia is not completely clear and may be related to insufficient hepatic synthesis of IGF-1. IGF-1 was significantly decreased in the younger sister, but not in the older sister, suggesting that growth retardation may result from a combination of causes; however, the safety and efficacy of GH in GSD Ia patients remains controversial^1^. Successful cases have been reported previously^[18]^; however, GH treatment may also increase the risk of liver adenoma, which can progress to hepatocellular carcinoma, a severe and fatal condition.^[19]^ The younger sister was observed after basic treatment, and, if necessary, GH therapy could be attempted under close monitoring.

Uncooked cornstarch therapy does not correct the underlying cause of disease and patients are at risk of serious long-term complications (e.g., hepatocellular adenomas, hepatocellular carcinoma, renal disease, and osteoporosis).^[1,20,21]^ Gene therapies in GSD Ia mouse models are promising. Gene therapy using AAV8 delivery vectors is currently being tested in ongoing clinical trials (NCT03517085 and NCT03970278). Other therapeutic approaches in preclinical development include mRNA therapy, gene editing, AAV9 gene therapy, and exon skipping.^[21]^ We look forward to the results of future studies.

In addition to the burden of disease, GSD patients also face social pressures. The proband in our report has dropped out of school and her younger sister is in elementary school. We tried to provide the proband patient with psychological support, but the damage caused by the illness made this less effective, so we hope that an increasing number of people will pay attention to such rare diseases and diagnose them in the early stage of the disease to avoid various serious consequences in adulthood and improve patient quality of life.

## 3. Conclusions

The patients conducted repeated visits for medical care, but no true cause was found for their condition. The proband’s recurrent pancreatitis caused portal hypertension and sepsis, which were life-threatening. There are still many challenges related to treatment of these diseases, and more attention should be paid to this rare disease.

## Acknowledgments

The authors are very grateful to the patient for his kind contribution to this study. We also would like to thank Editage (www.editage.cn) for English language editing.

## Author contributions

**Data curation:** Huilin Lu, Lu Yu.

**Conceptualization:** Shenglian Gan, Fang Yu.

**Writing—original draft:** Qin Liu.

**Writing—review and editing:** Shenglian Gan, Jian Luo, Ting Sun.
